# Heterogeneous trajectories of perceived stress and their associations with active leisure: a longitudinal study during the first year of COVID-19

**DOI:** 10.3389/fpubh.2024.1327966

**Published:** 2024-05-09

**Authors:** Karel Kulbin, Anna-Liisa Jõgi, Aleksander Pulver, Kristjan Kask

**Affiliations:** ^1^School of Natural Sciences and Health, Tallinn University, Tallinn, Estonia; ^2^School of Educational Sciences, Tallinn University, Tallinn, Estonia; ^3^Department of Teacher Education, University of Jyväskylä, Jyväskylä, Finland

**Keywords:** perceived stress, coping, active leisure, person-centered approach, COVID-19 pandemic

## Abstract

**Introduction:**

There is a plethora of literature on the dynamics of mental health indicators throughout the COVID-19 pandemic, yet research is scarce on the potential heterogeneity in the development of perceived stress. Furthermore, there is a paucity of longitudinal research on whether active leisure engagement, which typically is beneficial in reducing stress, might have similar benefits during times of major disruption. Here we aimed to extend previous work by exploring the dynamics of change in stress and coping, and the associations with active leisure engagement over the first year of COVID-19.

**Methods:**

Data from 439 adults (*M*_age_ = 45, SD = 13) in Estonia who participated in a longitudinal online study were analyzed. The participants were assessed at three timepoints: April–May 2020; November–December 2020; and April–May 2021.

**Results:**

Mean stress and coping levels were stable over time. However, latent profile analysis identified four distinct trajectories of change in stress and coping, involving resilient, stressed, recovering, and deteriorating trends. Participants belonging to the positively developing stress trajectories reported higher active leisure engagement than those belonging to the negatively developing stress trajectories.

**Discussion:**

These findings highlight the importance of adopting person-centered approaches to understand the diverse experiences of stress, as well as suggest the promotion of active leisure as a potentially beneficial coping resource, in future crises.

## 1 Introduction

It is accepted that COVID-19 and the circumstances surrounding the pandemic exacerbated mental health around the world. The COVID-19 pandemic spread in many waves, and this was accompanied by varying levels of social and economic restrictions and the accumulation of potentially stressful life circumstances (1). The pandemic outbreak constitutes an acute, large-scale, and uncontrollable stressor with a long-term impact. The detrimental impact of the pandemic on mental health has primarily been documented through the population-level increase in depression and anxiety symptoms (2, 3). The origin and the development of such mental health problems are consistently related to excessive stress [e.g., (4)], and these associations are aligned with the stress-vulnerability models of psychopathology (5, 6). These models explain the possible ways in which stressful experiences may trigger the onset of a mental health disorder, whether an individual is predisposed (i.e., vulnerable) to a mental health condition, and what role protective factors may play in these interactions. Numerous research evidence have linked high perceived stress not only to emotional disturbances such as anxiety (7) but also to physical health [e.g., hypertension, cardiovascular diseases (8)]. Identification of sub-populations with high risk of stress and interventions to reduce stress levels can potentially help to prevent later mental disorders (9).

The transactional stress model (10, 11) posits that a person’s capacity to cope and adjust to life challenges is a consequence of interactions that occur between a person and their environment. The ability to cope with stress depends on how an individual evaluates the relevance of the stressors (primary appraisal) and whether a person believes to hold sufficient resources to relieve or remove the stressor (secondary appraisal). In line with the transactional stress model, Cohen et al. (12) argue that a psychological state of perceived stress (hereafter stress) occurs when a situation in a person’s life is appraised as threatening or demanding and at the same time resources are insufficient to cope with the situation. However, this approach does not assume that certain life situations are inherently stressful but refers to the cognitive appraisal process where the cognitively mediated emotional response is given to the situation [e.g., (13)].

Several longitudinal studies among different age groups have investigated how stress levels may have changed during the pandemic. Most of these studies demonstrated a stable course of stress levels irrespective of the pandemic situation (14–16). Such findings have been explained considering the significant social and economic challenges (e.g., financial insecurities, changes in the working modalities, disruptions in the social life) that the pandemic brought in addition to the health crisis, and which together prolonged the risk of chronic stress. Salfi et al. (17) reported that stress levels even increased after the first lockdown period in the spring of 2020 and further plateaued by the second wave of the pandemic. They suggested that, in addition to a continuous societal and economic crisis, the lifting of restrictions in between the waves raised the perception of risk and thereby affected stress levels. Controversially, Gallagher et al. (18) demonstrated decreasing stress levels as the course of the pandemic continued and ascribed such findings to the presupposition that individuals become more resilient to the repercussions of the pandemic over time [see (19)].

Most of the previous longitudinal studies on the development of stress among adults during the COVID-19 pandemic have focused on the average changes (i.e., variable-centered approach), but the distinct courses of the stress over time (e.g., increasing for some, while decreasing for others) may bias the results and can obscure heterogeneous patterns (i.e., person-centered approach) of experiences. There is no reason to doubt that all the scenarios explained by the above-cited studies may have partly affected stress response throughout the pandemic but depended on many contextual and person-centered factors. A meta-analysis of the impact of COVID-19 on mental health indicators showed substantial heterogeneity among the findings of longitudinal studies (20), which suggests that there were no ubiquitous effects on mental health. Several longitudinal studies (21–23) have scrutinized the possibility of distinct courses of the change in symptoms of mental disorders (e.g., depression and anxiety). These studies found heterogeneous trajectories of symptoms during the pandemic, showing that approximately 70–80% of the population consistently reported no symptoms of mental disorders. They concluded that for smaller groups in the population symptoms of mental disorders increased, or in contrast decreased, as the pandemic continued its course. These findings add to evidence that lockdowns did not have evenly detrimental effects on mental health and that a certain proportion of people were psychologically resilient to the circumstances, or some might have even benefitted from the new work and life patterns.

A few studies have also employed a person-centered approach to examining perceived stress and stressor exposure based on cross-sectional data from the beginning of COVID-19. These studies have identified distinct profiles of pandemic-related exposure to stressors in adults (24) and heterogeneous profiles of stress and coping levels among pregnant women (25). However, such cross-sectional studies do not allow the examination of potentially distinct trajectory groups of stress developments over time. To our knowledge, the only published longitudinal investigation employing a person-centered approach for the examination of changes in perceived stress levels during the pandemic has been conducted among adolescents [age 12–15 years, (16)]. This study found no support for distinct trajectories of perceived stress. Adolescents (ages 12–15 years) were characterized by homogeneously stable and moderate stress levels during the first year of the pandemic. The authors explained this finding by assuming that adolescents commonly experience stress regardless of the pandemic, and thus pandemic-related stressors did not greatly affect their normative stress levels. To the best of our knowledge, no studies have scrutinized in adults the potential heterogeneity of trajectories (i.e., change over time) of perceived stress during the pandemic, and the findings of the previous variable-centered longitudinal studies on perceived stress are inconsistent (14, 15, 17, 18).

An all-embracing socio-historical event such as the COVID-19 pandemic provides a unique occasion to identify different paths of adaptation or maladaptation to persistent stressors and to examine coping resources that help individuals manage the effects of such drastic circumstances. Engagement in leisure activities is one such behavioral coping resource. Exploring how leisure contributes to relieving and counteracting stress has been studied for decades. Coleman and Iso-Ahola (26) first proposed in their theory that leisure facilitates social support and generates enduring beliefs of self-determination, which buffers the negative impact of stress on mental and physical health. In addition, Iwasaki and Mannell (27) described how leisure may act also as a strategy for palliative coping (i.e., temporarily diverting from stressful events to regroup and gain perspective) and mood enhancement (i.e., reducing negative mood and enhancing positive mood). Empirical studies have shown evidence that when people under stressful circumstances are engaged in leisure activities, the stress is reduced and therefore the negative impact of the stress on health is also reduced (28–31). Zawadzki et al. (32) have also identified the real-time within-person processes such that when individuals reported engaging in leisure, they had lower stress compared to when not engaged in leisure activity. Iwasaki (33, 34) has shown that leisure coping predicted positive coping outcomes even beyond the effects of general coping strategies (e.g., problem-focused coping unrelated to leisure).

Although no consensus definition of leisure engagement is imposed, prior research has mostly treated it as a behavioral concept—defined as the frequency or the amount of time in which one participates in leisure activities outside work duties, personal maintenance, and other obligations (35). The classification of leisure activities has neither been consistent in the literature. Leisure activities have been divided either as passive (also referred to as “low-demand” or “time-out” leisure) or active (also referred to as “high-demand” or “achievement” leisure) (36–38). Prior research has shown that engagement in active leisure activities (e.g., hobbies, physical, and nature-based activities) is more consistently linked with the benefits of stress reduction (30, 39, 40). Caltabiano (41) identified that outdoor activities/sports and hobbies were the most significant leisure activities to reduce stress. Such activities often involve using both physical and mental energy and often happen with other people. Iwasaki et al. (42) have emphasized that active leisure is more than just physical activities, and less physically active forms of leisure should not be undervalued in leisure coping processes. It can be assumed that active leisure activities involve ingredients (e.g., social interaction, creative expression, cognitive stimulation) to stimulate a wider range of mechanisms (e.g., psychological, biological, social) which may simultaneously play a role in alleviating stress [see (43)].

However, it has been shown that paradoxically people tend to reduce their participation in active leisure when they are stressed, which can be caused by an intuitive preference for passive leisure during hectic times or by a not deliberate reaction to the levels of stress (44). Thus, the relationship between active leisure and stress could be bidirectional, with stress also affecting motivation to engage in active leisure. At the same time, the options for active leisure were often restricted during the pandemic, possibly further limiting the engagement in active leisure. Previous studies have reported that the number of leisure activities people engaged in decreased (45), and engagement in physical and outdoor activities was reduced (46) during the first year of COVID-19.

Several studies have examined leisure engagement as a potential coping resource also during COVID-19. Based on the ecological momentary assessment data, it has been shown that engaging in free time was associated with lower stress levels during the pandemic (47). Existing findings also suggest that changes in leisure engagement (compared to pre-COVID) were related to poorer mental health (46, 48) and people who felt their current leisure engagement level fell below their desired level reported lower mental well-being (46). Takiguchi et al. (45) have shown in their longitudinal study that engaging in a larger number of leisure activities during the pandemic reduced depressive symptoms through resilience. However, longitudinal research is scarce on whether active leisure engagement, which is usually beneficial for stress reduction, might have similar benefits in times of major disruptions of the pandemic. It can be assumed that heterogeneous trajectories (if they emerged as such) of perceived stress during the pandemic were characterized by distinct levels of active leisure engagement. As engagement in active leisure is linked with the benefits of stress reduction (30, 39–41), it can be further assumed that higher engagement in active leisure was associated with positively developing (i.e., decreasing) stress trajectories. It can be expected that lower engagement in active leisure was related to negatively developing (i.e., increasing) stress trajectories.

The present study aims to explore the dynamics of change in stress and its associations with active leisure engagement as a stress coping resource over the first year of the COVID-19 pandemic. The study seeks to expand previous research by examining varying trajectories of change in stress (i.e., differences in the level, and the direction of change) and the interplay between the changes in stress and active leisure engagement over time. By doing this, we could gain a more differentiated understanding of the pandemic’s complex impact on stress levels and contribute to formulating guidance for stress-relieving behaviors in potential future lockdowns and pandemics.

As this study is exploratory by nature, to achieve the aim of the study, the following research questions are examined:

1. How did perceived stress change over the first year of COVID-19?
2. Can distinct trajectories be identified based on perceived stress levels over the first year of COVID-19?
3. How was active leisure engagement related to belonging to a certain stress trajectory over the first year of COVID-19?

## 2 Materials and methods

### 2.1 Procedure and sample

This study is part of a longitudinal investigation that focuses on the dynamics of mental health and well-being during the COVID-19 pandemic in Estonia. Approval for conducting the research was granted by the Tallinn University ethics committee (April 15, 2020; decision no 6). Voluntary participants were recruited for the survey via online ads (with the link to the survey) in news portals (e.g., Delfi.ee), and social media channels (e.g., Facebook). The entire study was conducted online using the SurveyMonkey platform. Estonian-speaking adults aged 18 or older currently residing in Estonia were eligible to participate. No compensation was offered as an incentive to participate. After reading an information page and confirming their informed consent, participants completed the survey. The datasets across three assessments were merged based on unique anonymized identification numbers (using SPSS). The data was collected over three timepoints across the first year of the COVID-19 pandemic: at Time 1 (T1 – April 20th until May 11th, 2020); at Time 2 (T2 – November 9th until December 6th, 2020); and at Time 3 (T3 – April 27th until May 23rd, 2021).

At T1, 530 participants were recruited for the longitudinal study, of whom 257 responded at T2 and 249 responded at T3. An additional 212 participants were recruited at T2 (via a similar strategy as at T1), of whom 142 responded also at T3. Two hundred participants responded to the survey at all three timepoints. To be able to analyze potential changes, those who had responded to the survey at least twice were included in the data analysis. This strategy resulted in a sample size of 448, which was predominantly composed of females (92.4%). Participants’ ages ranged from 18 to 81 (*M* = 45.37, SD = 12.97). 98.2% of the participants reported their native language as Estonian. In terms of relationship status, 31% were single (including widowed, divorced) and 69% were in a relationship (including married, cohabitation, civil partnership). 82.1% of the participants were employed, and 17.9% were not employed (including students, and pensioners).

**Figure 1:**
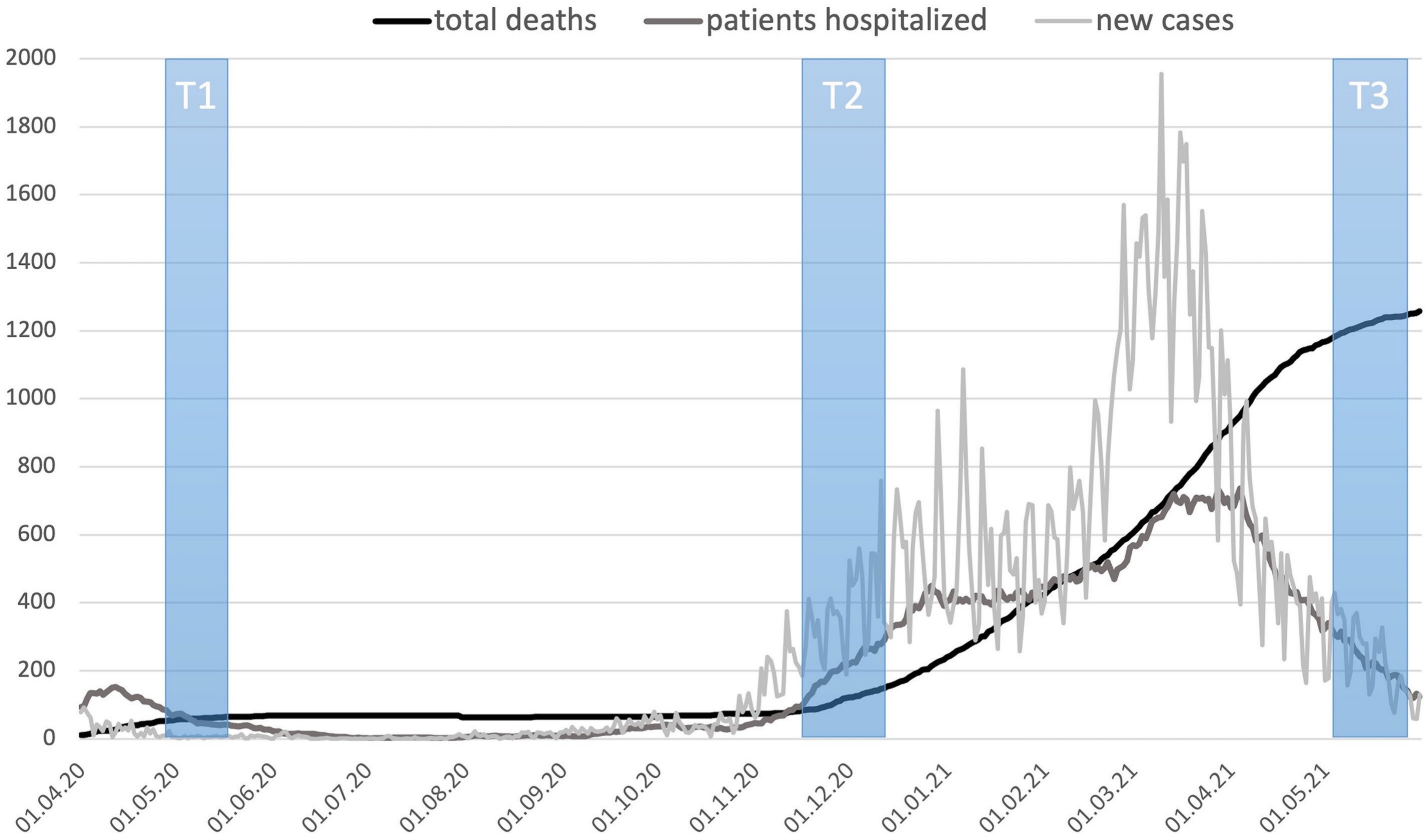
Situation during three data collection time windows (T1–T3): the number of COVID-19 deaths, patients hospitalized, and new cases per day. Source: Compiled by authors based on data provided by TEHIK (49).

Figure 1 shows the pandemic situation in Estonia during the three data collection periods. In spring 2020, while the first measurement (T1) occurred, the State of Emergency was in effect in Estonia, which meant that the availability of medical services was decreased. Students were transferred to distance learning; public gatherings were banned. Along with restrictions in traveling, all leisure facilities were closed, excluding parks and recreational trails if following the “2 + 2 rule” (i.e., a maximum of two people together at one time, keeping a minimum distance of two meters apart from others). In autumn 2020 (T2), after a relatively virus- and restriction-free summer, the second wave of the virus arrived, and the number of new cases was rising rapidly. However, by that time, lighter restrictions (compared to T1) were only being gradually re-introduced—schools were still open and leisure facilities were so far mostly available. The third data collection, in spring 2021 (T3), followed a period in which the numbers of new cases and hospitalizations had been the highest observed throughout the pandemic, and the government had re-imposed stricter restrictions lasting until May 2021. Widespread vaccination against COVID-19 in the general population (age groups below 60 years) did not start until mid-May 2021 in Estonia (49) when our third data collection (T3) was ending.

### 2.2 Measures

#### 2.2.1 Perceived stress

Perceived stress was assessed using the Estonian version of the 10-item Perceived Stress Scale [PSS-10; (50)]. Participants were asked to indicate on a five-point scale (0 = never, 4 = very often) how often they felt or thought a certain way during the last 4 weeks (e.g., “How often did you feel unable to control the important things in life?”). Originally, this self-reported questionnaire was designed to measure “the degree to which situations in one’s life are appraised as stressful” [(12), p. 385], consisting of six positively (items 1, 2, 3, 6, 9, 10) and four negatively (items 4, 5, 7, 8) worded items.

Although the scale was developed to capture stress as a single latent factor, following empirical studies in different contexts and languages using confirmatory factor analysis (CFA) techniques have predominantly shown that a two-factor model fits the data better than a unidimensional model (51–53). These two related factors have been described as (a) *perceived stress* (or helplessness; negatively worded items) and (b) *perceived coping* (or self-efficacy; positively worded items). In favor of the two-factor solution, authors have pointed out that the content of positively phrased and negatively phrased items do not coincide (54); and the two factors have shown distinct predictive qualities (55). For the Estonian version of the PSS-10, only the preliminary psychometric properties have been previously reported, based on principal component analysis and internal reliability coefficients for the unidimensional solution of the scale (56). Thus, CFA was conducted for the PSS-10 to examine whether a one- or two-factor solution fits the data best. Our data supported the two-factor model of the Estonian version of the PSS-10. Hence, the current study treated perceived stress as a two-dimensional construct of stress and coping. Longitudinal measurement invariance (MI) analysis was also conducted to ensure whether comparisons of stress and coping scores across the three timepoints were meaningful (57). Our data showed configural invariance and partial scalar and metric invariance in three timepoints, as factor loadings and item intercepts were allowed to vary for two items. The detailed results of CFA and MI are provided in Supplementary material. Cronbach’s alphas showed good internal consistency for both the stress and coping items at each time point (α = 0.83–0.88). Mean values were calculated for both scales at each timepoint and used in further analyses.

#### 2.2.2 Active leisure

Active leisure engagement was measured with a formative scale, comprising the frequency of respondents’ participation in three leisure activities: (1) *engaging in physical activities* (e.g., sports, walking); (2) *spending time in natural settings* (e.g., parks, forests); (3) *participating in main hobby/pursuit*. A similar aggregation approach has been used by numerous previous studies when the goal has been to capture a broader leisure activity domain with one indicator [e.g., (36, 46, 58)]. The three active leisure activities were selected based on literature: their stress-alleviating qualities have been widely described (30, 39, 40); and they have been consistently linked with better mental health, both before (59) and during the pandemic (48). Although our choice of leisure activity items was not all-inclusive, it tapped major active leisure engagement facets relevant to this study (39–43). Participants were asked to rate how often they spent time doing each of the activities during the last month. Response options were: 1 = “less than once a week or never”; 2 = “1–2 times a week”; 3 = “3–4 times a week”; 4 = “5–6 times a week”; 5 = “every day”; and 6 = “2 or more times a day.” The mean aggregation of the three activities was used, which weights each activity equally. Higher scores indicate higher active leisure engagement.

### 2.3 Statistical analyses

Since the final sample also included those participants who had missed one of the data collection points, the dataset had missing values of perceived stress, perceived coping, and active leisure engagement at different timepoints (31.7% of cases at T1; 10.9% at T2; 12.7% at T3). Regression imputation was used to preserve all cases and to fill in the missing values (60). For the imputation models of stress and coping, available scores of both constructs of the other two timepoints were used as predictors. In the regression imputation models for active leisure engagement, available scores of the other two timepoints of the same construct were used as predictors. Next, the stress and coping variables were scrutinized for the absence of multivariate outliers. Nine cases were eliminated as multivariate outliers, which resulted in the final sample size of 439. Further, repeated measures analysis of variance (ANOVA) controlling for covariates (age and gender) was used to examine changes in stress and coping over time. Violations of sphericity were addressed using Greenhouse–Geisser corrections.

Next, exploratory latent profile analysis (LPA) was used to identify distinct trajectories of perceived stress across the three timepoints. LPA as a person-centered technique allows the identification of heterogeneous subpopulations comprising distinct response patterns across time. Deciding the number of subgroups (i.e., trajectories) is based on the grouping precision and the comparative fit indices, as well as the interpretability of subgroups (61). Both stress and coping factors were modeled in one LPA with the variances allowed to vary between groups. Also, the covariance between stress in three timepoints and the covariance between coping in three time points were allowed to vary between groups. The fit of models with the different number of profiles was compared using the Akaike information criterion (AIC), the Bayesian information criterion (BIC), the sample-size adjusted Bayesian information criterion (aBIC), the Vuong-Lo-Mendell-Rubin likelihood ratio test (VMLR), bootstrapped likelihood ratio test (BLRT), a measure of entropy, interpretability of the observed trajectories, and the size of the profiles (61). After model selection, participants were classified according to their most likely profile membership.

Finally, a mixed ANOVA model controlling for age was run to examine the interaction between changes in active leisure engagement (time as a within-subjects factor) and trajectories of stress and coping (as a between-subjects factor). Gender was not included as a covariate due to the low number of men (<5) in some of the stress trajectory groups found with LPA. The assumption of homogeneity of variances was tested by Box’s M test. Violations of sphericity were corrected by applying a Greenhouse–Geisser correction. For *post hoc* multiple comparisons, Bonferroni adjustment was used.

CFA and invariance tests were performed in R version 4.1.3 (62), using *lavaan* package (63). Regression imputations were performed in R package *mice* (64). LPA was conducted using Mplus 8.8 (65). ANOVAs were performed in SPSS version 28.

## 3 Results

The means, standard deviations, ranges, Cronbach’s alphas, and bivariate correlations for all the study variables are shown in Table 1.

**Table 1:** Means (M), standard deviations (SD), ranges, Cronbach’s alphas (α), and correlations between study variables.

	*M*	SD	Range	*α*	1	2	3	4	5	6	7	8
1. Perceived stress T1^a^	1.75	0.76	0.17–4	0.86	–							
2. Perceived stress T2^a^	1.74	0.79	0–4	0.88	0.54	–						
3. Perceived stress T3^a^	1.73	0.80	0–4	0.88	0.52	0.70	–					
4. Perceived coping T1^a^	2.53	0.66	0–4	0.87	−0.71	−0.50	−0.50	–				
5. Perceived coping T2^a^	2.49	0.71	0–4	0.83	−0.42	−0.72	−0.59	0.61	–			
6. Perceived coping T3^a^	2.48	0.74	0–4	0.86	−0.37	−0.55	−0.71	0.66	0.70	–		
7. Active leisure engagement T1^b^	3.34	1.07	1–6	–	−0.19	−0.22	−0.21	0.23	0.19	0.20	–	
8. Active leisure engagement T2^b^	3.00	1.12	1–6	–	−0.13	−0.23	−0.25	0.17	0.21	0.22	0.70	–
9. Active leisure engagement T3^b^	3.17	1.15	1–6	–	−0.14	−0.26	−0.27	0.17	0.23	0.28	0.64	0.69

*N* = 439; all correlations were statistically significant at *p* < 0.01.

a Scale from 0 to 4.

b Scale from 1 to 6.

### 3.1 Changes in stress and coping: variable-centered approach

First, changes in average perceived stress and perceived coping during the first year of the pandemic were investigated using repeated measures ANOVA. There were no significant changes found across three timepoints in mean scores of stress, *F* (1.88, 818.60) = 0.83, *p* = 0.43, *η_p_*^2^ = 0.002, nor in mean scores of coping, *F* (2, 872) = 0.84, *p* = 0.43, *η_p_*^2^ = 0.002.

### 3.2 Distinct trajectories of stress and coping: person-centered approach

To identify potential distinct trajectories of stress during the first year of the pandemic, a latent profile analysis was conducted on perceived stress and coping scores measured at three timepoints. Six sets of LPA-s were compared. The drop of AIC and aBIC values decelerated, and BIC value did not further decrease, after the four-trajectory solution (see Table 2 for the fit indices, entropy, and group sizes). The five-trajectory solution did not reveal any new patterns of change, and the more parsimonious four-trajectory model was chosen as it had the best interpretability.

**Table 2:** Fit statistics for comparison of different longitudinal latent profile models of perceived stress and coping.

Number of profiles	*N*	AIC	BIC	aBIC	Entropy	VLMR *p*-value	BLRT *p*-value
1	439	4,886	4,959	4,902	–	–	–
2	191/248	4,624	4,775	4,658	0.74	0.04	<0.01
3	215/120/104	4,522	4,751	4,573	0.76	0.16	<0.01
**4**	**66/120/144/109**	**4,426**	**4,732**	**4,494**	**0.78**	**0.03**	**<0.01**
5	104/70/52/85/128	4,363	4,747	4,448	0.81	0.27	<0.01
6	83/105/94/28/73/56	4,339	4,800	4,442	0.82	0.76	0.14

N, group sizes; AIC, Akaike information criterion; BIC, Bayesian information criterion; aBIC, sample-size adjusted Bayesian information criterion; VLMR, Vuong-Lo-Mendell-Rubin likelihood ratio test; BLRT, bootstrapped likelihood ratio test. Estimates of the chosen four-trajectory solution are bolded.

**Figure 2:**
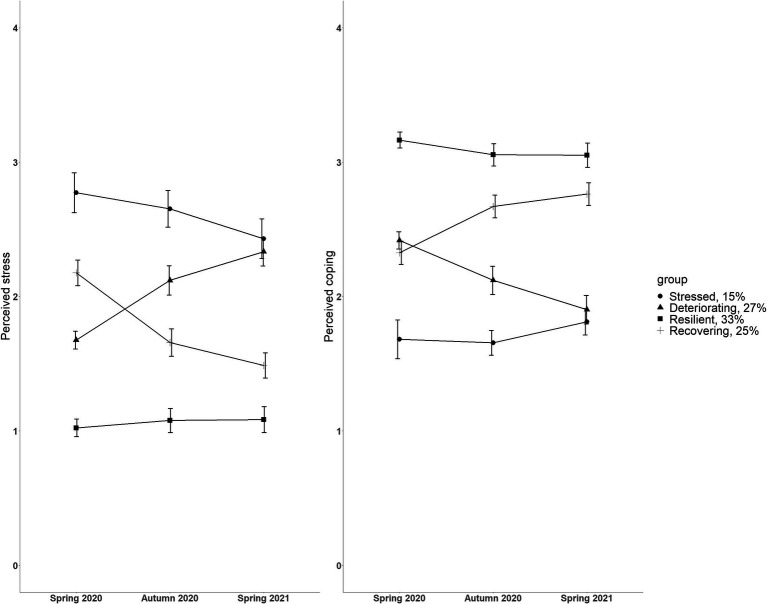
Estimated mean perceived stress (left) and perceived coping (right) scores from the four-trajectory solution of the latent profile analysis across three timepoints. Each group indicates a distinct trajectory during the first year of the pandemic. Both scales from 0 to 4. Error bars represent 95% confidence intervals.

Figure 2 presents the stress and coping trajectories over three timepoints for four groups identified in the LPA model. The first trajectory, labeled as ‘*Stressed*’ (15%, *N* = 66), was characterized by a high stress level and a low coping level throughout the study. In the second trajectory, labeled as “*Deteriorating*” (27%, *N* = 120), the participants had relatively low stress and high coping at the beginning of the pandemic, but it was followed by a sustained incline in stress and decline in coping throughout the first year of the pandemic. The largest proportion of participants (33%, *N* = 144) belonged to the third trajectory labeled as “*Resilient*.” The participants in this group had consistently low stress and high coping across the first year of the pandemic. In the fourth trajectory, labeled as “*Recovering*” (25%, *N* = 109), the participants reported relatively high levels of stress at the beginning of the pandemic. However, these participants “bounced back” over time, as indicated by a decline in stress and an incline in coping throughout the next two timepoints.

Next, we tested if the four groups identified in the LPA were characterized by differences in age, relationship status, or work status. A one-way ANOVA was used to compare the mean age between four stress and coping groups. There was no statistically significant difference in age between the four groups [*F* (3, 435) = 2.36, *p* = 0.07]. Chi-square tests were used to examine if the group membership was related to relationship status or work status. Relationship status was dichotomized into “single” (incl. Widowed, divorced) and “in a relationship” (incl. Married, cohabitation, civil partnership). Work status was dichotomized into “employed” and “not employed” (incl. Student, pensioner). Group membership was neither related to relationship status [*X*^2^ (3, *N* = 439) = 1.39, *p* = 0.71] nor to work status [*X*^2^ (3, 439) = 3.57, *p* = 0.31].

### 3.3 Changes in active leisure engagement in relation to distinct trajectories of stress and coping

**Figure 3:**
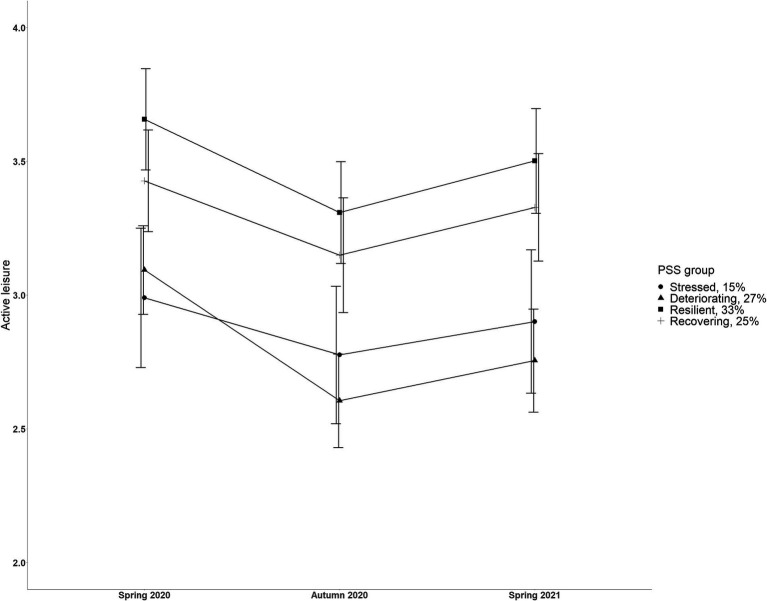
Estimated mean active leisure engagement scores across three timepoints according to four distinct stress trajectories. Scale from 1 to 6. Error bars represent 95% confidence intervals.

Changes in active leisure engagement were investigated in relation to distinct trajectories of stress and coping. Specifically, a 4 (trajectories) X 3 (timepoints) mixed ANOVA model controlling for age was run to examine the interaction effect between trajectories of stress and coping (group membership as a between-subjects factor) and time (as a within-subjects factor) on active leisure engagement. The mixed ANOVA results are illustrated in Figure 3.

There was a main effect of time on active leisure engagement, *F* (1.96, 850.85) = 9.68, *p* < 0.001, *η_p_*^2^ = 0.02. Bonferroni adjusted pairwise comparisons showed a quadratic effect such that active leisure engagement decreased (*p* = 0.001) from spring 2020 to autumn 2020, and then increased (*p* = 0.001) from autumn 2020 to spring 2021 (see Table 1 for means and SDs). There was a main effect of stress and coping trajectory membership on active leisure engagement, *F* (3, 434) = 12.18, *p* < 0.001, *η_p_*^2^ = 0.08. Bonferroni adjusted pairwise comparisons revealed that participants belonging to the “Resilient” (*M* = 3.46, SD = 0.94) trajectory reported higher active leisure engagement than those in the “Stressed” (*M* = 2.93, SD = 0.93) and “Deteriorating” (*M* = 2.83, SD = 0.93) trajectories (both comparisons *p* < 0.001). In addition, participants belonging to the “Recovering” (*M* = 3.30, SD = 0.93) trajectory reported higher active leisure engagement than those in the “Deteriorating” (*M* = 2.83, SD = 0.93) trajectory (*p* < 0.001). The interaction between the stress trajectories and changes in active leisure engagement was not found, *F* (5.88, 850.85) = 1.30, *p* = 0.26, *η_p_*^2^ = 0.009, failing to prove that changes in active leisure engagement were related to distinct trajectories of stress and coping.

## 4 Discussion

The present study aimed to explore the dynamics of change in stress and its associations with active leisure engagement as a stress coping resource during the first year of the COVID-19 pandemic. Our person-centered analytical approach with longitudinal data adds to previous research by identifying heterogeneous trajectories of change in stress among adults. In addition, the current study extends previous research by demonstrating how stress trajectories were characterized by distinct levels of active leisure engagement in times of major social and economic disruptions of the pandemic.

Addressing the first research question, the results from variable-centered analyses indicated that perceived stress and coping levels were stable irrespective of the situation over the first year of the pandemic. Such finding coincides with many of the longitudinal studies on stress levels during the pandemic (14–16). However, as our subsequent person-centered analyses showed, the depiction obtained through the conventional variable-centered approach failed to capture the complexity of the situation.

Our second research question aimed at identifying potentially distinct stress trajectories. The person-centered (latent profile) analyses, based on perceived stress and coping scores measured at three timepoints, revealed a more nuanced understanding of temporal stress dynamics during the first year of the pandemic among adults. Four heterogeneous trajectories of change in stress and coping were identified.

The largest proportion of the sample belonged to the *Resilient* group (33%), with consistently stable low stress and high coping across the year. This group was composed of individuals who tended to appraise the circumstances as not harmful for them and/or perceived their resources as sufficient to cope with the demands, regardless of the varying conditions throughout the first year of the pandemic (11, 66). The clear emergence of such a group also supports Bonanno’s (67) work on arguing how a substantial proportion of individuals endure aversive events with minor effects on their healthy functioning.

One-quarter of the sample consisted of *Recovering* individuals, who experienced relatively high levels of stress during the first spring of the pandemic, but “bounced back” during the following year. This favorable adaptation trajectory could be ascribed to novelty, unpredictability, and initial difficulties with new obligations that caused acute stress during the first wave of the virus, but over time adaptation to the conditions occurred and the situation was appraised as less threatening [see (19, 68)].

Over a quarter of our sample belonged to the *Deteriorating* trajectory, with relatively low stress and high coping at the beginning of the pandemic which was followed by a sustained incline in stress and decline in coping over the study period. A continuous societal and economic crisis, loss of hope for a quick end to the pandemic, and a possible increase in perception of health risks (17) may have played a role for the individuals in the deteriorating trajectory.

The smallest proportion of our sample belonged to the *Stressed* group, who experienced high stress and low coping levels throughout the study period. Since we did not possess pre-pandemic data on our sample, it is not possible to credibly attribute high stress levels to the pandemic. Nevertheless, these patterns of increasing or persistently excessive stress levels call for particular attention.

Our analyses demonstrated that focusing only on the average changes (i.e., variable-centered approach) obscures the variability of the temporal changes in stress during the pandemic and could lead to oversimplified inferences. As opposed to the assumption of uniform effects of the varying circumstances of the pandemic on stress levels (14, 17, 18), our study highlights that there was a clear heterogeneity of temporal changes in perceived stress across the first year of the pandemic. More generally, this means that the identification of different subgroups in the temporal process of stress provides an opportunity to describe differences in the details of effective coping with stress. Contrary to our results, a study conducted among adolescents found no evidence of heterogeneity in stress trajectories during the first year of the pandemic (16). We assume that the different target populations of these studies explain the discrepancy in findings. One possible explanation is that changes in the daily routine of adults were more heterogeneous compared to adolescents (e.g., interruptions in the typical school routines were similar for all students). Among adults, previous studies on mental disorder symptoms during the pandemic that employed a person-centered approach, have consistently shown distinct trajectories of the symptoms’ development (21–23) and thus, support our findings considering the link between stress and psychopathology (6). Interestingly, the four trajectories also overlap with the prototypical outcome trajectories of human stress responses after potentially traumatic life events [see (68)]. It seems that continuous and potentially stressful conditions of the pandemic (i.e., chronic events) were followed by a similar heterogeneity of stress responses across time, as have been observed after short-term aversive life events (i.e., acute events). When considering the socio-demographics potentially associated with the four stress trajectories, our analysis indicated that the distinct trajectories could not be attributed to age, being single (vs. in a relationship), or being employed (vs. not employed). This partially contradicts previous findings which have consistently shown that younger age is related to a higher risk for negatively developing mental health trajectories (21–23, 68).

Addressing our third and final research question, active leisure engagement across three timepoints was investigated in relation to distinct trajectories of stress and coping. We found that the data collection period had a small effect on average active leisure engagement levels. Even though in the autumn of 2020 there were fewer restrictions on leisure activities than in the rest of the data collection periods, in the autumn of 2020 our study participants were less engaged in active leisure, compared to the spring of 2020 or the spring of 2021. Thus, this finding can be attributed rather to a seasonal effect, as inclement and uncomfortable weather conditions in north temperate zones in autumn have been shown to reduce the frequency of active leisure engagement (69).

The participants’ active leisure engagement levels were found to differ according to their stress trajectories membership irrespective of the timepoint of assessment. As it was assumed, participants belonging to the positively developing stress trajectories reported higher active leisure engagement than those belonging to the negatively developing stress trajectories (specifically, *Resilient* compared to *Stressed* and *Deteriorating*; *Recovering* compared to *Deteriorating*). Thus, our findings not only support existing studies (30, 39, 40, 47) but also extend previous studies by indicating a potentially preventive effect of active leisure engagement on perceived stress during times of crises (while options for leisure are often limited). Importantly, we cannot rule out the possibility of a bidirectional relationship between stress levels and active leisure engagement. It has been previously shown that perception of stress may negatively affect motivation to engage in active leisure (44). The interaction effect between the changes in active leisure engagement across time and the stress trajectories was not found in our study, indicating that distinct developments in stress during the pandemic were not attributable to the addition of, or shrinkage in, active leisure. However, a slight tendency toward such an effect was noticeable (Figure 3), where individuals in the *Deteriorating* stress trajectory tended to decrease their active leisure engagement between spring and autumn 2020 more than individuals in other trajectories; and it warrants attention in future research.

## 5 Limitations

Despite our contributions to a better understanding of the complex temporal dynamics of stress and its longitudinal associations with active leisure engagement in times of major social and economic disruptions, our research has several limitations. First, based on our observational data, we cannot be sure of the direction of associations, and intervention studies are needed to infer causality. A clear limitation is the absence of pre-pandemic data on our sample that would have facilitated a more detailed interpretation of the stress trajectories, and their associations with active leisure engagement. Caution should be taken when interpreting stress levels as ‘due to the pandemic’ since such a supposition remains speculative. Second, we cannot rule out self-selection bias that may have occurred using an online survey; health-conscious people may have been more interested in participating in a mental health study. Most concerning is the underrepresentation of males (7.6%) in our sample. Thus, we must be especially careful when making inferences about men. Challenges with male recruitment are widely documented, especially in online public health surveys (70). Still, we could not overcome this issue because the data collection needed to be urgently started to study this unpredictable period of the pandemic. Third, additional person-related confounders (e.g., health status, contracting the virus, job insecurity, social support, personality traits) and environmental factors such as season might have influenced our findings. These variables were not included in our study, and we recommend accounting for them in future research. Finally, a rather broad measure of active leisure engagement (i.e., aggregation of three activities) was used in our study, and future research should consider scrutinizing the possible differential and additive effects of specific leisure activities.

## 6 Conclusion

Our findings indicate substantial variabilities in the level and in the direction of change in stress during an all-embracing socio-historical crisis. The study highlights the importance of considering individual differences in stress appraisal and adopting person-centered approaches to understand the diverse experiences of stress and coping during future crises. Heterogeneous trajectories of perceived stress were characterized by distinct levels of active leisure engagement. Our findings extend previous studies by pointing to the stable link between higher active leisure engagement and lower perceived stress during the pandemic while options for active leisure were often limited. We highlight the importance of promoting and facilitating opportunities for active leisure as a potentially beneficial coping resource during times of crisis. As male participants were underrepresented in our study, special caution should be taken when generalizing the findings to men.

## Data availability statement

The raw data supporting the conclusions of this article will be made available by the authors, without undue reservation.

## Ethics statement

The study involving humans was approved by Tallinn University ethics committee. The study was conducted in accordance with the local legislation and institutional requirements. The participants provided their written informed consent to participate in this study.

## Author contributions

KKu: Conceptualization, Data curation, Formal analysis, Investigation, Methodology, Writing – original draft, Writing – review & editing, Visualization. A-LJ: Conceptualization, Data curation, Formal analysis, Visualization, Writing – review & editing. AP: Conceptualization, Methodology, Writing – review & editing. KKa: Conceptualization, Funding acquisition, Investigation, Methodology, Project administration, Writing – review & editing.
