# Supplementary material for: Heterogeneous trajectories of perceived stress and their associations with active leisure: a longitudinal study during the first year of COVID-19
Source: Front Public Health. 2024 May 9;12:1327966. doi: 10.3389/fpubh.2024.1327966 (PMC11112114; doi:10.3389/fpubh.2024.1327966)
Supplement: Supplementary file 1 [file Table_1.DOCX]

Supplementary material. Confirmatory factor analysis and longitudinal measurement invariance analysis for the Estonian version of the PSS-10.

The confirmatory factor analysis (CFA) with maximum likelihood robust estimator was conducted for the PSS-10 to examine whether a one- or two-factor solution fits the data best. Starting with the data from T1, the one-factor model was first estimated, which did not fit the data well [*χ^2^* (35) = 226.80, p < 0.001; comparative fit index (CFI) = 0.84; Tucker-Lewis (TLI) = 0.79; root mean square error of approximation (RMSEA) = 0.13; standardized root mean square residual (SRMR) = 0.06]. Then the two-factor model with correlation allowed between the factors was tested. Based on the modification indices, one correlation was added between residuals of two similar items of the *perceived coping* factor. A good fit of the two-factor model to the data was obtained [*χ^2^* (33) = 76.05, p < 0.001; CFI = 0.97; TLI = 0.95; RMSEA = 0.07; SRMR = 0.04]. The standardized correlation coefficient between the two factors (*perceived stress* and *perceived coping)* was 0.78, and all factor loadings were ≥ 0.6. In summary, data supported the two-factor model of the Estonian version of the PSS-10. Hence, the current study treated stress as a two-dimensional construct of perceived stress and perceived coping.

The longitudinal measurement invariance analysis for the PSS-10 was conducted, to ensure whether comparisons between three timepoints are meaningful. Using the nested models, configural invariance (i.e., the factor structure is the same across time points), metric invariance (i.e., factor loadings are the same across time points), and scalar invariance (i.e., item intercepts are the same across time points) were examined (57). Our data showed configural invariance and partial metric and scalar invariance in three timepoints as factor loadings and item intercepts were allowed to vary for two items. Model fit comparisons are provided in the table below.

Comparison of configural, metric, and scalar invariance models of the PSS-10 by three timepoints

| Model | χ^2^ | *df* | CFI | TLI | RMSEA | SRMR | χ^2^ difference test |
| --- | --- | --- | --- | --- | --- | --- | --- |
| Configural | 593.20* | 357 | .96 | .95 | .04 | .04 | - |
| Metric | 624.73* | 373 | .96 | .95 | .04 | .05 | χ2 (16) = 32.11, *p* = .01 |
| Partial metric | 612.46* | 369 | .96 | .95 | .04 | .05 | χ2 (12) = 19.28, *p* = .08 |
| Partial scalar | 633.03* | 381 | .96 | .95 | .04 | .05 | χ2 (12) = 20.62, *p* = .06 |

Note. χ2 = Satorra–Bentler scaled chi-square test statistic; df = degrees of freedom; CFI = comparative fit index; TLI = Tucker-Lewis index; RMSEA = root mean square error of approximation; SRMR = standardized root mean square residual. In χ2 difference tests models are compared to previous model, except Partial metric is compared to Configural. *p<0.001.
